# Murine Models of Splenic Marginal Zone Lymphoma: A Role for *Cav1*?

**DOI:** 10.3389/fonc.2016.00258

**Published:** 2016-12-14

**Authors:** Chelsey L. Patten, Christine E. Cutucache

**Affiliations:** ^1^University of Nebraska at Omaha, Omaha, NE, USA

**Keywords:** splenic marginal zone lymphoma, 7q minimally deleted region, 7q LOH, genetically engineered, neoplasms

## Abstract

Dozens of murine models of indolent and aggressive B-cell lymphomas have been generated to date. These include those manifesting chronic lymphocytic leukemia (CLL), diffuse large B-cell lymphoma (DLBCL), as well as xenografts of mantle cell lymphoma (MCL). These models have led to an improved understanding of disease etiology, B-cell biology, immunomodulation, and the importance of the tumor microenvironment. Despite these efforts in CLL, DLBCL, and MCL, considerably little progress toward a model of splenic marginal zone lymphoma (SMZL) has been accomplished. Herein, we describe the similarities and differences between CLL, MCL, and SMZL and highlight effective murine models that mimic disease in the two former, in hopes of informing a potential model of the latter. At the time of writing this review, the precise molecular events of SMZL remain to be determined and a treatment regimen remains to be identified. Therefore, based on the efforts put forth in the B-cell lymphoma field throughout the past three decades, the established role of *caveolin-1* in B- and T-cell biology as an oncogene or tumor suppressor, and the recurrent deletion or loss of heterozygosity (LOH) of 7q in many cancers, we make recommendations for a murine model of SMZL.

## Background

Splenic marginal zone lymphoma (SMZL) is a newly acknowledged cancer that effects primarily middle-aged and elderly patients. This disease primarily effects patients ages 60 and older without evidence of gender predominance (1). Since SMZL is less than a quarter-century old in terms of recognition by the World Health Organization, little progress has been made regarding disease pathogenesis.

Although multiple B-cell lymphoma murine models have been created, currently there is no mouse model dedicated specifically to understanding the disease pathology of SMZL. With no definitive clues as to what drives this disease, the development of a reliable and replicable *in vivo* model is needed in order to understand the basic oncogenic factors leading to SMZL. In this review, we discuss possible contributing factors in tumorigenesis, highlight three similar B-cell malignancies [i.e., chronic lymphocytic leukemia (CLL), mantle cell lymphoma (MCL), and SMZL]—including their shared immunophenotype, as well as currently existing murine models of these neoplasms—and finally we discuss the role for 7q in SMZL. Ultimately, we make a recommendation for the generation of a murine model with a knockout at 7q involving *caveolin-1 (CAV1)*, based on its role in related B-cell malignancies.

## Possible Contributing Factors Leading to Tumorigenesis

When designing a mouse model that can accurately recapitulate SMZL, it is imperative to understand all of the factors that may contribute to the tumorigenesis of the disease. To shed light on which factors specifically contribute to the occurrence of SMZL, a population-based study was conducted in the United States on a cohort from 2001–2008 on SMZL incidence and patient survival. Several possible contributing factors emerged from this study, including autoimmune disease (20% of cases), environmental factors, and aging-related effects, such as chronic inflammation, DNA damage, and a diminished immune response (1, 2). In addition to these, specific factors that might influence oncogenic events can include regulatory elements, infection, genetic mutations (transcriptomic), or epigenetics (methylation patterns).

Differences in disease drivers can distinguish SMZL into two different subtypes. Approximately half of SMZL cases are likely caused by infectious disease, more specifically, the hepatitis C virus [HCV; (3)]. This infectious disease-driven subtype suggests that virally transformed animal models might be an effective way to study disease onset and progression. However, chromosomal aberrations are present in the vast majority (>70%) of all SMZL cases, implying that a genetic component may serve as the major driver of tumorigenesis (4). Clinically, these two subtypes of SMZL are distinct in terms of disease progression and prognosis for patients.

### Infections

Many studies suggest an antigenic role for tumorigenesis of SMZL. In fact, 30–40% of cases have shown to be disease driven (5). The possible causative role of HCV has been suggested both based on high prevalence as well as multiple epidemiologic and therapeutic studies (1, 3, 6). The etiology of HCV and its relation to B-cell lymphomas in general has been correlated to the geographic distribution in a few studies with small case numbers, mainly in Japan and Southern Italy. Overall, local HCV prevalence along with genetic and environmental factors may play a part in the geographically diverging results (3). Although geographic distribution has not yet been shown to have a significant impact on prevalence rates, an increase in HCV-positive persons from 2.7 million to 3.2 million from 1994–1998 to 1999–2002 mirrored increased prevalence rates of SMZL during this same time frame (2). Furthermore, from 2001 to 2008, the incidence of SMZL continued to steadily climb (2) and now comprises 2% of all lymphoid neoplasms (7). Although the exact pathogenesis mechanism is unknown, both chronic antigen stimulation and viral lymphotropism may contribute to progression of the malignant cell (3).

Findings that intraclonal diversification caused by ongoing somatic hypermutation was identified in 81% of the rearrangements using the *IGHV1*-2*04 genes versus only 40% of rearrangements using other *IGHV* genes. This supports the idea of antigen selection in SMZL ontogeny, as well as the possibility of ongoing antigen involvement throughout the progression of the disease, even possibly toward diffuse large B-cell lymphoma (DLBCL) like suggested in the “Multistep Theory of Lymphomagenesis” (7–10). The 14q32 band holds IGHV, and translocations involving the 14q32 band have been detected less frequently in SMZL than in non-Hodgkin lymphomas. Half of SMZL patients carry an increased load of IGHV somatic mutations, which is associated with improved prognosis (11). Combined, this supports the argument that IGHV1-2*04 SMZL is, in fact, a distinct molecular SMZL subtype that needs to be recognized and studied *in vivo* (8–11).

### Genetic Mutations

Although many SMZL cases have shown a possibility for a disease-driven etiology, more than 70% of SMZL cases show some form of chromosomal aberration, most predominantly a loss of heterozygosity (LOH) at 7q (2). This 7q LOH is observed in 40–50% of total cases, and the 7q31–32 deletion is present in approximately 45% of all cases, serving as the most common cytogenetic abnormality, thus suggesting a genetic driver (8, 9, 12–16). Many studies have been done in hopes of finding exactly which genes and pathways are altered in SMZL, therefore serving as potential targets on the development of SMZL murine models. A summary of these studies can be found in Table 1 (8, 9, 11, 13–15, 17–25). Although many genes (most notably NOTCH2, KLF2, KLF4, and BIRC3) have been reported as mutated in SMZL, we suggest that the 7q deletion is of primary importance, as it is possible this deletion serves as a marker for disease progression and may even be a causative event, rather than a pro-survival function as was previously speculated (9).

**Table 1:** **Summary of reported genomic mutations or deletions in splenic marginal zone lymphoma (SMZL) (8, 9, 11, 13–15, 17–25)**.

Study	Genes	Prominent pathways
Arcaini et al. (23)	IGH, CDKN21, TRAF5, REL, PKCA	NF-κB pathway
Novak et al. (19)	TNFAIP3 (A20)	NF-κB pathway, toll-like receptor responses
Rinaldi et al. (24)	POT1, TP53, TNFAIP3 (A20), BCL6, NFKBIZ	NF-κB pathway, toll-like receptor responses, TP53 pathway
Salido et al. (26)	IGH, PAX5, IGK, IGL, ATM, POT1, SHH, MIR17HG, TP53, TNFAIP3 (A20)	Apoptotic signaling, cell cycle regulation, TP53 pathway
Fresquet et al. (22)	PAX5, MIR17HG, TP53, IRF5, TNPO3, RBM28, SND1, TMEM209, CALU, COPG2, IMPDH1, CPA4, LRRC4, MYC, ZC3HC1, CDK6, CCND3, NFKB2, SOX5, RHOH, L324N, BCL6, BCL2, MALT1, CDKN21, MIR182	TP53 pathway, information-processing pathway at the IFN-beta enhancer, MAPK signaling, cyclins and cell cycle regulation, proliferation, anti-apoptotic
Kiel et al. (18)	MLL2, MLL3	Chromatin remodeling and transcriptional regulation
Rossi et al. (17)	TP53, NOTCH2, NOTCH1, SPEN, DTX1, SWAP70, EGR2, EGR1, IKBKB, TRAF 3, TNFAIP3 (A20), MAP3K14, MYD88, BIRC3, CD79A, CARD11, TBL1XR1, SIN3A, EP300, ARID1A, TRRAP, GPS2, MCRS1, MSL2, HIST1H2AG, HIST1H2BK, WAC, MLL2	NOTCH pathway, TP53 pathway, NF-κB pathway, toll-like receptor responses, MAP KINASE, BCR pathway, anti-apoptotic, chromatin remodeling and transcriptional regulation
Watkins et al. (21)	IRF5, TNPO3, KLHDC10, RBM28, NIPA, SND1, TSGA14, TMEM209, UBE2H, NRF1, NAG8, ATP6V1F, CALU, AHCYL2, COPG2, HIG2, TSPAN33, IMPDH1, CPA4, OPN1SW, LRRC4, CPA5, CPA2, TSGA13, CPA1, C7orf45, NYD-SP18	Information-processing pathway at the IFN-beta enhancer
Arribas et al. (11)	TP53, IRF5, NOTCH2, BIRC3, RHOH, BCL2, TCL1A, SYK, BLNK, NFATC1, MUM1, AIM2, IL2RA, IL7, IL6, IL21, BCL2L10, FOXP1, CD40, CD70, TNFRSF9, TAC1, CD44, IL2RA, MMP12, MEST, MET, PTN, FCGR3A, FCGR3B, TNFAIP2, TNFAIP1, CXCL1, CXCL2, TGFBI, ICAM2, PIP5K2A, EIF2AK, MMP9	NOTCH pathway, information-processing pathway at the IFN-beta enhancer, TP53 pathway, proliferation, anti-apoptotic, NF-κB pathway, BCR signaling, interleukin, integrins TNF signaling, growth receptor factor signaling, B-cell function, cell adhesion, kinase, inhibition of protein metalloproteinases
Parry et al. (20)	TNFAIP3 (A20), MAP3K14, MLL2, AMOTL1, FAT4, FBXO11, PLA2G4D, TRRAP, USH2A, CBFA2T, CREBBP, CACNA1E, CACNAIH, CACNA2D2, FLNC, MAPK8IP3, RASA1, TAOK3, PIWIL3, NOTCH4, MAML3, CULI1, CDC27, FLT1, CRLF2, EZH2, CBFA2T3, ZNF434	NF-κB Pathway, toll-like receptor responses, MAP kinase, chromatin remodeling and transcriptional regulation, multistep regulation of transcription by Pitx2, the PRC2 complex sets long-term gene silencing through modification of histone tails, actions of nitric oxide, VEGF, cell cycle regulation
Piva et al. (8)	NOTCH2	NOTCH pathway
Arribas et al. (15)	NOTCH2, CACNB2, HTRA1, KLF4	Notch pathway
Clipson et al. (25)	TP53, TRAF3, TNFAIP3 (A20), CARD11, KLF2	NF-κB pathway, toll-like receptor responses, BCR pathway, TP53 pathway
Parry et al. (9)	IGH, TP53, NOTCH2, TNFAIP3 (A20), MYD88, ARID1A, MLL2, CREBBP, BCL6, KLF2, P53	NOTCH pathway, TP53 pathway, NF-κB pathway and toll-like receptor responses, cell cycle, anti-apoptotic
Peveling-Oberhag et al. (13)	NOTCH2, MYD88, KLF2, SMYD1, GRIN2C, CDC27, HERC2, APOA4, CSMD1, PRSS1, PCLO, PDE10A, ZNF451, ZNF608, FBXO44, LOC728888, CACNA1C, KRTAP5-2, STMN4, MUC12, POM121, BTN2A2, SLC6A7, TTC14, CEBPZ	NF-κB pathway, toll-like receptor responses, NOTCH1 signaling, nitric oxide signaling, synaptic protein signaling, MAPK signaling
Arcaini et al. (14)	NOTCH2, KLF2	NOTCH pathway

### Methylation Patterns

In a genome-wide DNA-promoter methylation profiling study by Arribas et al., two main clusters were distinguished based on the degree of promoter DNA methylation (15). This high-M cluster not only had an inferior outcome and showed high risk for histologic transformation to DLBCL but suggests that DNA hypermethylation could act together with 7q31-32 deletion, NOTCH2 mutation, and IGHV1-02, to determine a distinct genetic and epigenetic subgroup of SMZL (11).

## Birds of a Feather: B-Cell Neoplasms CLL, MCL, and SMZL

**Figure 1:**
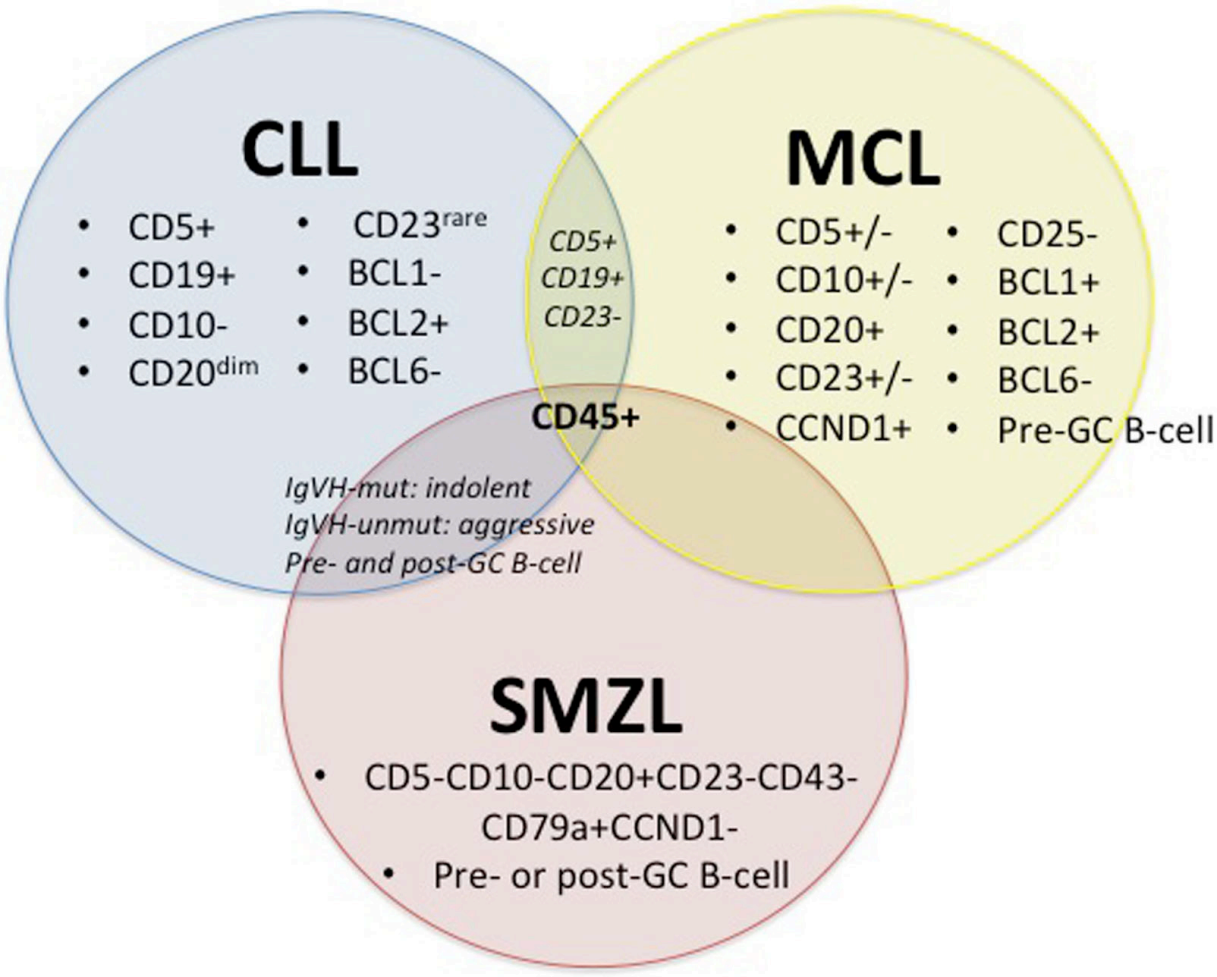
**Diagnostic factors of CLL, MCL, and SMZL**. +/−, rarely expressed; CLL, chronic lymphocytic leukemia; MCL, mantle cell lymphoma; SMZL, splenic marginal zone lymphoma (27–30).

Chronic lymphocytic leukemia, MCL and SMZL are all neoplasms that affect mature B-cells; yet, in contrast to its B-cell counterparts, SMZL is the least studied. These three malignancies have major commonalities (27–30), including some shared surface markers, shared pathways for disease progression (15), and hypothesized infectious disease drivers, including HCV (Figure 1).

Figure 1 depicts immunophenotypical similarities and differences across CLL, MCL, and SMZL. The expression of CD5, CD19, BCL2, and rarely CD23 along with the absence of CD10 and BCL1 with diminished CD20 set CLL apart from its close relatives. However, the presence of CD5 and CD19 and the absence of CD23 can also indicate MCL, so it is important to run a multitude of panels in order to solidify a diagnosis. MCL can display variable expression of CD5, CD10, and CD23 and express CD20, CCND1, BCL1, and BCL2, while lacking CD25 and BCL6. SMZL expresses CD20 and CD79a but lacks CD5, CD10, CD23, CD43, and CCND1. Both CLL and SMZL are thought to originate from pre- or post-germinal center B-cells. This etiology differs from that of MCL, which is known to arise from pre-germinal center B-cells. Last, CLL and SMZL have a common prognostic factor with mutated *IGVH*, leading to improved outcome for patients (27–30).

## Existing Murine Models

The development and maintenance of murine models are important due to (i) difficulty in gaining access to patient samples, (ii) inability to study host:tumor interactions in an *ex vivo* system, and (iii) lack of cell lines that adequately recapitulate human disease (31). Both CLL and MCL have been well-characterized, including having murine models developed, while a dedicated, specific SMZL murine model driven by a transgene is still absent. Although previous SMZL models have been attempted, they often lead to development of DLBCL and, therefore, do not serve as true SMZL models (since clinically when SMZL progresses to DLBCL, additional transcriptomic events occur). The current murine models for CLL and MCL, as well as those identified to-date for SMZL are discussed below.

### Chronic Lymphocytic Leukemia

Chronic lymphocytic leukemia is the most common adult leukemia in the Western world, and consequently, in comparison to its B-cell counterparts, murine models for CLL are the most well-defined. This is in part due to their ability to accurately recapitulate human disease, specifically the tumor microenvironment that is paramount for the maintenance of CLL cells. Because of this, it is imperative that CLL must harbor in supportive organs both in patients and in mouse models (31, 32). The most accurate recapitulation of CLL *in vivo* is arguably the Eμ-*TCL1* transgenic mouse from the C. Croce lab (31), and this argument is emphasized in a review by Bresin et al. (33). Conversely, the NZB-*IRF4*^−^*^/^*^−^ model from R. Lu’s lab is reminiscent of the aggressive phenotype of CLL (34). The NZB-*IRF4*^−/−^ mice developed spontaneous CLL at 100% penetrance – thereby indicating a relationship between levels of *IRF4* and CLL development. In these mice, CLL cells with V_H_11 proliferated predominantly in the spleen, thus allowing this model to serve as a successful study agent for molecular pathogenesis as well as for therapeutic techniques (35).

While the Eμ-*TCL1* and NZB-*IRF4*^−^*^/^*^−^ lines most accurately recapitulate human CLL, cell line xenografts have also been established. Kellner and colleagues developed the first long-term proliferative cell line for CLL from a (del)17p case and furthered this development by using this cell line to create a xenograft model that recapitulates CLL (31, 36). These are major strides in CLL research, as no other group has been able to create a reliable, actively replicating CLL cell line. Other examples include xenografts of peripheral blood CLL and MCL cells in alymphoid mice by other labs (37, 38) thoroughly reviewed by Chen and Chiorazzi (39).

Not surprisingly, as CLL is the most common adult leukemia and receives the greatest amount of work by clinicians and scientists alike, major strides have been made to create reliable murine models for disease (including both the indolent and aggressive phenotypes).

### Mantle Cell Lymphoma

Mantle cell lymphoma is an aggressive, yet rare, neoplasm that affects less than 200,000 people in the United States annually (10). In comparison to CLL cells, MCL cells are far less reliant on tumor microenvironment. Therefore, mouse models of MCL have largely come from xenografts that poorly mimic tumor microenvironmental conditions; however, MCL cells are less reliant on tumor microenvironment than CLL cells. By utilizing the method of tail vein injection, *in vivo* models of metastatic human MCL were established as described herein, briefly. Both primary cell lines from MCL cases and MCL cell lines (including Jeko-1, Mino, Rec-1, Hbl-2, and Granta-519) were used to generate disease in immunodeficient NOD.Cg-*Prkdc^scid^ Il2rg^tm1Wgl^*/Szj mice or NOD.SCID mice (40–42). Hegde et al. (41) and Ahrens et al. (40) in the S. S. Joshi’s lab created therapy-resistant models to study the impact of novel adjuvants for improving patient outcome. These xenografts were effective; namely, 7 out of 12 patient samples allowed for engraftment observation with the use of primary MCL cell lines, while all 5 of the MCL cell lines showed tumor engraftment. The overall survival of these mice varied with regards to the specific cell line used but ranged from 22 ± 1 to 54 ± 3 days (41, 42). Furthermore, the NOD.Cg-*Prkdc^scid^ Il2rg^tm1Wgl^*/Szj MCL mouse model has been shown to aid in testing systemic chemotherapy, monoclonal antibodies, and angiogenesis inhibitors, which will serve as a significant tool in understanding this disease and advancing diagnostic and therapeutic techniques. Moreover, the NOD/SCID IL2Rgamma-null xenograft model was effective for studying *p53*-mutated CLL and *ATM-*mutated MCL (38).

Taken together, these studies have led to recapitulation of MCL (both from primary cells and cell lines) *in vivo*, thereby providing effective murine models to use for drug testing as well as to increase the understanding of basic disease biology and disease progression.

### Splenic Marginal Zone Lymphoma

Like MCL, SMZL is a rare neoplasm. Due to its relatively recent identification, it is not surprising that many questions linger about its oncogenesis and pathogenesis. SMZL has been observed spontaneously in a few murine models, namely, *NFS.N* mice expressing murine leukemia virus (5, 43, 44) as well as a transcription factor-induced transgenic line with murine leukemia virus (45). Although the latter model develops SMZL, it quickly progresses into full-blown DLBCL. Similarly, the loss of a potent tumor suppressor, *p53*, leads to a SMZL-like phenotype in mice (46). All of these mouse models aiming to recapitulate human SMZL *in vivo* include a viral component or a major genomic hit that is non-reminiscent of the human presentation.

An *in vivo* murine model to mimic SMZL remains to be created, but because of its potential at assisting hematologists in understanding basic B-cell biology, germinal center reactions, and ultimately tumorigenesis, a new model is most certainly warranted. The recent identification of the role for the 7q31-32 region, a common minimally deleted region in human cancers, reignites the efforts to pursue causative mutations in a coding or non-coding region within that locus.

## LOH AT 7q31: Potential for Murine Model Development to Mimic SMZL

There are many regions of the genome considered “fragile” sites, and the long arm of chromosome 7 is one such region. Some studies have mapped the specific portions of this region that show causality with tumorigenesis, but others have simply recognized the region as a whole. LOH is observed at 7q31-34 in the following cancers: SMZL, meningioma, papillary thyroid carcinoma, ovarian cancer, acute myeloid leukemia, myelodysplastic syndrome, and pancreatic carcinoma. Deletions at 7q have been reported in a variety of human neoplasias including leukemia, breast, ovary, colon, prostate, gastric, head and neck, pancreatic, and renal cell carcinomas (47).

Several groups have sought a “master regulator” within the commonly deleted 7q region (and corresponding downregulated genes) in severe cases of SMZL. However, despite valiant efforts at well-controlled experiments (9), a master regulator has yet to be identified. The development of such a model to explore driver mutations leading to SMZL would shed light on SMZL pathophysiology as well as biological behavior, thereby serving as an effective model.

### A Role for *Cav1*

Fragile site FRA7G holds two tumor suppressor genes, including *CAV1* and *TES*. Cav1 has been shown to serve as an oncogene or tumor suppressor depending on cell or tissue type, microenvironmental influence, or disease progression (48). CAV1 7q31.2 deletions/translocations appear to be downregulated in ovarian, lung, and mammary tumors while upregulated in prostate, bladder, thyroid, and esophageal carcinomas (47). Haploinsufficiency of *CAV1* is enough to induce partial transformation of human breast epithelial cells (49). Additionally, the loss of *Cav1* combined with MMTV-PyMT leads to the acceleration of mammary tumors (47). *Cav1-*knockout mice do not spontaneously develop tumors, although the skin is more susceptible to chemical carcinogenic treatment. Specifically, when *Cav1*^−^*^/^*^−^ mice were exposed to 7,12-dimethylbenz(a) anthracene, epidermal cells significantly upregulated *CCND1* and *ERK1/2* and mice developed hyperplastic ductal epithelium (47, 50).

In addition to carcinogenic challenges in *Cav1*-null mice, recent studies identify a link between *Cav1* and both B- and T-cell neoplasms. Specifically, *CAV1* was identified as a major immunoregulator, which was significantly upregulated in aggressive CLL cases (48). When Eμ-*TCL1-Tg-Cav1*^−^*^/^*^−^ mice were generated, they displayed a significantly more aggressive phenotype than the indolent Eμ-*TCL1* model (51). Moreover, the role of *Cav1* is well-documented in immune synapse formation (48, 52), thereby suggesting another contribution to immune evasion and tumorigenesis. Most recently reported, *CAV1* is deleted in up to 45% of SMZL cases (16). Additionally, *CAV1* has been documented to play a key role as a potential diagnostic marker in T-cell malignancies as it is significantly upregulated in more than 67% of T-cell malignancies (53). Taken together, these data suggest a clear role for *CAV1* in both B- and T-cell neoplasms. Therefore, as *CAV1* is lost or mutated in up to half of all SMZL cases and the 7q region (where *CAV1* is located) is commonly deleted, we suggest that a murine model under an oncogenic driver, coupled with *Cav1* LOH, could be an effective model.

## Summary

*CAV1* resides at the 7q region and is heterogeneously expressed in cancer. Specifically, *CAV1* is downregulated in SMZL cases, while upregulated in aggressive cases of chronic lymphocytic leukemia and five types of mature T-cell lymphomas. We (and others) have demonstrated a critical role for *CAV1* in immune synapse formation, cellular proliferation, and cellular migration. *Cav1*^−^*^/^*^−^ mice are immunosuppressed, but the mechanism by which this immune dysregulation occurs is currently unknown. Improving our understanding of the basic biology of *CAV1* in the immune response will translate to (i) determining its role in the immune response governing antigen-presentation events in healthy and diseased immune responses and (ii) how *CAV1* controls immunophenotype/immune cell frequency. We have shown *CAV1* to be significantly dysregulated across more than 67% of cases of mature T-cell lymphomas as well as in aggressive cases of chronic lymphocytic leukemia. Recent data from others identify the 7q deletion in another mature B-cell lymphoma: SMZL. Therefore, results generated from this proposal would be applicable across lymphoid neoplasms. Taken together, our preliminary data support a hypothesized role for *CAV1* in lymphoid tumor progression, likely through an immune regulatory method.

Based on the knockout studies involving *p53*, coupled with the high incidence of spontaneous Hodgkin’s lymphoma development on the Swiss Jim Lambert mice, we suggest the development of a SMZL murine model with *Cav1* LOH with or without an oncogenic driver, such as *p53*^−^*^/^*^−^ or *MYC-Tg*. Finally, the *CAV1* deletion being present in up to 45% of all SMZL cases suggests that generating a *MYC-Tg-Cav1*^−^*^/^*^−^ mouse could serve as a more representative clinical mimic of SMZL. Based on the known role of *CAV1* in aggressive cases of both B- and T-cell malignancies, its role in immune regulation through immune synapse formation, its role in the tumor microenvironment, and the frequency of LOH at 7q, we suggest that this deletion could serve as a marker for disease progression and may even be a causative event.

## Author Contributions

Both CP and CC conceptualized, wrote, and edited the manuscript and approved this final version.

## Conflict of Interest Statement

The authors declare that the research was conducted in the absence of any commercial or financial relationships that could be construed as a potential conflict of interest.

## References

[1] Matutes E Oscier D Montalban C Berger F Callet-Bauchu E Dogan A Splenic marginal zone lymphoma proposals for a revision of diagnostic, staging and therapeutic criteria. Leukemia (2008) 22:487–95. 10.1038/sj.leu.2405068 18094718

[2] Liu L Wang H Chen Y Rustveld L Liu G Du XL Splenic marginal zone lymphoma: a population-based study on the 2001–2008 incidence and survival in the United States. Leuk Lymphoma (2013) 54(7):1380–6. 10.3109/10428194.2012.743655 23101590

[3] Sulyok M Makara M Újhelyi E Vályi-Nagy I Non-Hodgkin lymphoma and hepatitis C: where we are and what next? Pathol Oncol Res (2014) 21:1–7. 10.1007/s12253-014-9845-z 25273531

[4] Baliakas P Strefford JC Bikos V Parry M Stamatopoulos K Oscier D. Splenic marginal-zone lymphoma: ontogeny and genetics. Leuk Lymphoma (2015) 56(2):301–10. 10.3109/10428194.2014.919636 24798744

[5] Franco V Florena AM Iannitto E. Splenic marginal zone lymphoma. Blood (2003) 101:2464–72. 10.1182/blood-2002-07-2216 12446449

[6] Vannata B Stathis A Zucca E. Management of the marginal zone lymphomas. Cancer Treat Res (2015) 165:227–49. 10.1007/978-3-319-13150-4_9 25655612

[7] [SEER] Surveillance, Epidemiology, and End Results Program. National Institutes of Health. (2016). Available from: http://seer.cancer.gov/seertools/hemelymph/51f6cf57e3e27c3994bd5327/

[8] Piva R Deaglio S Famà R Buonincontri R Scarfò I Bruscaggin A The Krüppel-like factor 2 transcription factor gene is recurrently mutated in splenic marginal zone lymphoma. Leukemia (2014) 29(2):503–7. 10.1038/leu.2014.294 25283840

[9] Parry M Rose-Zerilli MJ Ljungström V Gibson J Wang J Walewska R Genetics and prognostication in splenic marginal zone lymphoma: revelations from deep sequencing. Clin Cancer Res (2015) 21(18):4174–83. 10.1158/1078-0432.CCR-14-2759 25779943 PMC4490180

[10] Bikos V Karypidou M Stalika E Baliakas P Xochelli A Sutton LA An immunogenetic signature of ongoing antigen interactions in splenic marginal zone lymphoma expressing IGHV1-2*04 receptors. Clin Cancer Res (2016) 22(8):2032–40. 10.1158/1078-0432.CCR-15-1170 26647217

[11] Arribas JA Gómez-Abad C Sánchez-Beato M Martinez N DiLisio L Casado F Splenic marginal zone lymphoma: comprehensive analysis of gene expression and miRNA profiling. Mod Pathol (2013) 26(7):889–901. 10.1038/modpathol.2012.220 23429603

[12] Tsieh S Flow Cytometry, Immunohistochemistry, and Molecular Genetics for Hematologic Neoplams. Philadelphia: Lippincott Williams & Wilkins, a Wolters Kluwer Business (2012).

[13] Peveling-Oberhag J Wolters F Döring C Walter D Sellmann L Scholtysik R Whole exome sequencing of microdissected splenic marginal zone lymphoma: a study to discover novel tumor-specific mutations. BMC Cancer (2015) 15:773. 10.1186/s12885-015-1766-z 26498442 PMC4619476

[14] Arcaini L Rossi D Paulli M Splenic marginal zone lymphoma: from genetics to management. Blood (2016) 127(17):2072–81. 10.1182/blood-2015-11-624312 26989207

[15] Arribas AJ Rinaldi A Mensah AA Kwee I Cascione L Robles EF DNA methylation profiling identifies two splenic marginal zone lymphoma subgroups with different clinical and genetic features. Blood (2015) 125(12):1922–31. 10.1182/blood-2014-08-596247 25612624 PMC4416938

[16] Ruiz-Ballesteros E Mollejo M Rodriguez A Camacho FI Algara P Martinez N Splenic marginal zone lymphoma: proposal of new diagnostic and prognostic markers identified after tissue and cDNA microarray analysis. Blood (2005) 106(5):1831–8. 10.1182/blood-2004-10-3898 15914563

[17] Rossi D Trifonov V Fangazio M Bruscaggin A Rasi S Spina V The coding genome of splenic marginal zone lymphoma: activation of NOTCH2 and other pathways regulating marginal zone development. J Exp Med (2012) 209(9):1537–51. 10.1084/jem.20120904 22891273 PMC3428941

[18] Kiel MJ Velusamy T Betz BL Zhao L Weigelin HG Chiang MY Whole-genome sequencing identifies recurrent somatic NOTCH2 mutations in splenic marginal zone lymphoma. J Exp Med (2012) 209(9):1553–65. 10.1084/jem.20120910 22891276 PMC3428949

[19] Novak U Rinaldi A Kwee I Nandula SV Rancoita PM Compagno M The NF-{kappa}B negative regulator TNFAIP3 (A20) is inactivated by somatic mutations and genomic deletions in marginal zone lymphomas. Blood (2009) 113(20):4918–21. 10.1182/blood-2008-08-174110 19258598 PMC2686142

[20] Parry M Rose-Zerilli MJ Gibson J Ennis S Walewska R Forster J Whole exome sequencing identifies novel recurrently mutated genes in patients with splenic marginal zone lymphoma. PLoS One (2013) 8(12):e83244. 10.1371/journal.pone.008324 24349473 PMC3862727

[21] Watkins AJ Hamoudi RA Zeng N Yan Q Huang Y Liu H An integrated genomic and expression analysis of 7q deletion in splenic marginal zone lymphoma. PLoS One (2012) 7(9):e44997. 10.1371/journal.pone.0044997 23028731 PMC3441634

[22] Fresquet V Robles EF Parker A Martinez-Useros J Mena M Malumbres R High-throughput sequencing analysis of the chromosome 7q32 deletion reveals IRF5 as a potential tumour suppressor in splenic marginal-zone lymphoma. Br J Haematol (2012) 158(6):712–26. 10.1111/j.1365-2141.2012.09226.x 22816737

[23] Arcaini L Lazzarino M Colombo N Burcheri S Boveri E Paulli M Splenic marginal zone lymphoma: a prognostic model for clinical use. Blood (2006) 107(12):4643–9. 10.1182/blood-2005-11-4659 16493005

[24] Rinaldi A Mian M Chigrinova E Arcaini L Bhagat G Novak U Genome-wide DNA profiling of marginal zone lymphomas identifies subtype-specific lesions with an impact on the clinical outcome. Blood (2010) 117(5):1595–604. 10.1182/blood-2010-01-264275 21115979

[25] Clipson A Wang M de Leval L Ashton-Key M Wotherspoon A Vassiliou G KLF2 mutation is the most frequent somatic change in splenic marginal zone lymphoma and identifies a subset with distinct genotype. Leukemia (2015) 29(5):1177–85. 10.1038/leu.2014.330 25428260

[26] Salido M Baro C Oscier D Stamatopoulos K Dierlamm J Matutes E Cytogenetic aberrations and their prognostic value in a series of 330 splenic marginal zone B-cell lymphomas: a multicenter study of the Splenic B-cell Lymphoma Group. Blood (2010) 116(9):1479–88. 10.1182/blood-2010-02-267476 20479288

[27] Matutes E Morilla R Owusu-Ankomah K Houlihan A Catovsky D. The immunophenotype of splenic lymphoma with villous lymphocytes and its relevance to the differential diagnosis with other B-cell disorders. Blood (1994) 83(6):1558–62. 8123845

[28] Armitage JO Weisenburger DD. New approach to classifying non-Hodgkin’s lymphomas: clinical features of the major histologic subtypes. Non-Hodgkin’s lymphoma classification project. J Clin Oncol (1998) 16(8):2780–95. 9704731 10.1200/JCO.1998.16.8.2780

[29] Savilo E Campo E Mollejo M Pinyol M Piris MA Zukerberg LR Absence of cyclin D1 protein expression in splenic marginal zone lymphoma. Mod Pathol (1998) 11(7):601–6. 9688179

[30] Ghia P Guida G Stella S Gottardi D Geuna M Strola G The pattern of CD38 expression defines a distinct subset of chronic lymphocytic leukemia (CLL) patients at risk of disease progression. Blood (2003) 101(4):1262–9. 10.1182/blood-2002-06-1801 12406914

[31] Bichi R Shinton SA Martin ES Koval A Calin GA Cesari R Human chronic lymphocytic leukemia modeled in mouse by targeted TCL1 expression. Proc Natl Acad Sci U S A (2002) 99:6955–60. 10.1073/pnas.102181599 12011454 PMC124510

[32] Kurtova AV Balabrishnan K Chen R Ding W Schnabl S Quiroga MP Diverse marrow stromal cells protect CLL cells from spontaneous and drug-induced apoptosis: development of a reliable and reproducible system to assess stromal cell adhesion-mediated drug resistance. Blood (2009) 114:4441–50. 10.1182/blood-2009-07-233718 19762485 PMC4081374

[33] Bresin A D’Abundo L Narducci MG Fiorenza MT Croce CM Negrini M TCL1 transgenic mouse model as a tool for the study of therapeutic targets and microenvironment in human B-cell chronic lymphocytic leukemia. Cell Death Dis (2016) 7:e2071. 10.1038/cddis.2015.419 26821067 PMC4816192

[34] Ma S Shukla V Fang L Gould KA Joshi SS Lu R. Accelerated development of chronic lymphocytic leukemia in New Zealand Black mice expressing a low level of interferon regulatory factor 4. J Biol Chem (2013) 288(37):26430–40. 10.1074/jbc.M113.475913 23897826 PMC3772189

[35] Shukla V Ma S Hardy RR Joshi SS Lu R. A role for IRF4 in the development of CLL. Blood (2013) 122(16):2848–55. 10.1182/blood-2013-03-492769 23926303 PMC3798999

[36] Kellner J Wierda W Shpall E Keating M McNiece I Isolation of a novel chronic lymphocytic leukemic (CLL) cell lines and development of an in vivo mouse model of CLL. Leuk Res (2016) 40:54–9. 10.1016/j.leukres.2015.10.008 26601610 PMC11770973

[37] Oldreive CE Skowronska A Davies NJ Parry H Agathanggelou A Krysov S T-cell number and subtype influence the disease course of primary chronic lymphocytic leukaemia xenografts in alymphoid mice. Dis Model Mech (2015) 8(11):1401–12. 10.1242/dmm.021147 26398941 PMC4631786

[38] Verner J Trbusek M Chovancova J Jaskova Z Moulis M Folber F NOD/SCID IL2Rγ-null mouse xenograft model of human p53-mutated chronic lymphocytic leukemia and ATM-mutated mantle cell lymphoma using permanent cell lines. Leuk Lymphoma (2015) 56(11):3198–206. 10.3109/10428194.2015.1034701 25827173

[39] Chen SS Chiorazzi N. Murine genetically engineered and human xenograft models of chronic lymphocytic leukemia. Semin Hematol (2014) 51(3):188–205. 10.1053/j.seminhematol.2014.05.001 25048783

[40] Ahrens AK Chaturvedi NK Nordgren TM Dave BJ Joshi SS. Establishment and characterization of therapy-resistant mantle cell lymphoma cell lines derived from different tissue sites. Leuk Lymphoma (2012) 53(11):2269–78. 10.3109/10428194.2012.691481 22568512

[41] Hegde GV Nordgren TM Munger CM Mittal AK Bierman PJ Weisenburger DD Novel therapy for therapy-resistant mantle cell lymphoma: multipronged approach with targeting of hedgehog signaling. Int J Cancer (2012) 131(12):2951–60. 10.1002/ijc.27602 22511234

[42] Klanova M Soukup T Jaksa R Molinsky J Lateckova L Maswabi BCL Mouse models of mantle cell lymphoma, complex changes in gene expression and phenotype of engrafted MCL cells: implications for preclinical research. Lab Invest (2014) 94:806–17. 10.1038/labinvest.2014.61 24862967

[43] Tang JC Ho FC Chan AC Chow EY Srivastava G. Progression of spontaneous lymphomas in SJL mice: monitoring in vivo clonal evolution with molecular markers in sequential splenic samples. Lab Invest (1998) 78:1459–66. 9840620

[44] Fredrickson TN Lennert K Chattopadhyay SK Morse HC III Hatley JW Splenic marginal zone lymphomas of mice. Am J Pathol (1999) 154:805–12. 10.1016/S0002-9440(10)65327-8 10079258 PMC1866400

[45] Hough MR Reis MD Singaraja R Bryce DM Kamel-Reid S Dardick I A model for spontaneous B-lineage lymphomas in IgHmu-HOX11 transgenic mice. Proc Natl Acad Sci U S A (1998) 95:13853–8. 10.1073/pnas.95.23.13853 9811890 PMC24927

[46] Ward JM Tadesse-Heath L Perkins SN Chattopadhyay SK Hursting SD Morse HC III. Splenic marginal zone B-cell and thymic T-cell lymphomas in p53-deficient mice. Lab Invest (1999) 50:3–14. 9952106

[47] Drusco A Pekarsky Y Costinean S Antenucci A Conti L Volinia S Common fragile site tumor suppressor genes and corresponding mouse models of cancer. J Biomed Biotechnol (2011) 4(6):1–10. 10.1155/2011/984505 21318118 PMC3035048

[48] Gilling CE Mittal AK Chaturvedi NK Iqbal J Aoun P Bierman PJ Lymph node-induced immune tolerance in chronic lymphocytic leukaemia: a role for caveolin-1. Br J Haematol (2012) 158(2):216–31. 10.1111/j.1365-2141.2012.09148.x 22571278

[49] Zou W McDanela L Smith LM Caveloin-1 haploinsufficiency leads to partial transformation of human breast epithelial cells. Anticancer Res (2003) 23(6):4581–6. 14981899

[50] Lee H Park DS Razani B Russel RG Pestell RG Lisanti MP Caveolin-1 mutations (P132L and null) behaves in a dominant-negative manner and caveolin01 (-/-) null mice show mammary epithelial cell hyperplasia. Am J Pathol (2002) 161(4):1357–69. 10.1016/S0002-9440(10)64412-4 12368209 PMC1867297

[51] Shukla A Cutucache CE Sutton GL Pitner MA Rai K Rai S Absence of caveolin-1 leads to delayed development of chronic lymphocytic leukemia in Eµ-TCL1 mouse model. Exp Hematol (2016) 44(1):30.e–7.e. 10.1016/j.exphem.2015.09.005 26435347

[52] Tomassian T Humphries LA Liu SD Silva O Brooks DG Miceli MC. Caveolin-1 orchestrates TCR synaptic polarity, signal specificity, and function in CD8 T cells. J Immunol (2011) 187(6):2993–3002. 10.4049/jimmunol.1101447 21849673 PMC3881976

[53] Herek TA Shew TD Spurgin HN Cutucache CE. Conserved molecular underpinnings and characterization of a role for caveolin-1 in the tumor microenvironment of mature T-cell lymphomas. PLoS One (2015) 10(11):e0142682. 10.1371/journal.pone.0142682 26566034 PMC4643970

